# Influence of bacterial *N*-acyl-homoserine lactones on growth parameters, pigments, antioxidative capacities and the xenobiotic phase II detoxification enzymes in barley and yam bean

**DOI:** 10.3389/fpls.2015.00205

**Published:** 2015-04-10

**Authors:** Christine Götz-Rösch, Tina Sieper, Agnes Fekete, Philippe Schmitt-Kopplin, Anton Hartmann, Peter Schröder

**Affiliations:** ^1^Research Unit Environmental Genomics, Helmholtz Zentrum München – German Research Center for Environmental Health, NeuherbergGermany; ^2^Research Unit Analytical BioGeoChemistry, Helmholtz Zentrum München – German Research Center for Environmental HealthNeuherberg, Germany

**Keywords:** AHL, *N*-acyl-homoserine lactone, glutathione *S*-transferase, antioxidant enzymes, barley, yam bean, quorum sensing, rhizosphere

## Abstract

Bacteria are able to communicate with each other and sense their environment in a population density dependent mechanism known as quorum sensing (QS). *N*-acyl-homoserine lactones (AHLs) are the QS signaling compounds of Gram-negative bacteria which are frequent colonizers of rhizospheres. While cross-kingdom signaling and AHL-dependent gene expression in plants has been confirmed, the responses of enzyme activities in the eukaryotic host upon AHLs are unknown. Since AHL are thought to be used as so-called plant boosters or strengthening agents, which might change their resistance toward radiation and/or xenobiotic stress, we have examined the plants’ pigment status and their antioxidative and detoxifying capacities upon AHL treatment. Because the yield of a crop plant should not be negatively influenced, we have also checked for growth and root parameters. We investigated the influence of three different AHLs, namely *N*-hexanoyl- (C6-HSL), *N*-octanoyl- (C8-HSL), and *N*-decanoyl- homoserine lactone (C10-HSL) on two agricultural crop plants. The AHL-effects on *Hordeum vulgare* (L.) as an example of a monocotyledonous crop and on the tropical leguminous crop plant *Pachyrhizus erosus* (L.) were compared. While plant growth and pigment contents in both plants showed only small responses to the applied AHLs, AHL treatment triggered tissue- and compound-specific changes in the activity of important detoxification enzymes. The activity of dehydroascorbate reductase in barley shoots after C10-HSL treatment for instance increased up to 384% of control plant levels, whereas superoxide dismutase activity in barley roots was decreased down to 23% of control levels upon C6-HSL treatment. Other detoxification enzymes reacted similarly within this range, with interesting clusters of positive or negative answers toward AHL treatment. In general the changes on the enzyme level were more severe in barley than in yam bean which might be due to the different abilities of the plants to degrade AHLs to metabolites such as the hydroxy- or keto-form of the original compound.

## Introduction

Since about 480 million years, when plants started to conquer dry land, they had to arrange life in a close relationship with soil microorganisms in the rhizosphere. In the interface with the soil, they had to cope with microbes colonizing their roots and were thus also regularly exposed to bacterial signaling substances. It was long unknown that bacteria possess the ability to communicate; they were rather considered as “deaf, blind, and solitary" (Obst, 2007) until the “Quorum Sensing" (QS) was described (Fuqua et al., 1994; Bassler, 2002; Galloway et al., 2011). Since then, the QS phenomenon has attracted considerable interest in the scientific community. In short, bacteria constitutively produce signal compounds at very low rates which are diluted in the environment unless the concentration of Homoserine lactone (AHL)-producing bacteria is high enough for the signal substances to accumulate and reach a certain threshold inside the cell to trigger a response. After binding to a specific receptor protein to form a complex in the cytosol of bacteria, expression or repression of certain genes such as for the formation of biofilms, production of antibiotics, plasmid transfer, bioluminescence, and several more is initiated (reviewed in Turovskiy et al., 2007). According to recent findings, QS not only enables bacteria to sense their quorum, but also spatial distribution parameters and may therefore be better described as a way to maximize the efficiency of bacterial actions that could be termed “efficiency sensing" (Hense et al., 2007).

We have now reached a substantial level of knowledge on QS, but we must bear in mind that bacteria live in complex environments exposed to species of all taxa, which may also communicate with each other in different ways. What if these languages would interfere with each other? This question has already been addressed early in the Tower of Babel story which vividly depicts the importance of trouble-free communication in an association of different individuals. One of the closest associations in this context is the one between bacteria and plants, resulting in various kinds of interplay from mutualism over a benign co-existence up to parasitic diseases. We already know quite well that this kind of interplay between plants and bacteria and their signal AHLs exists. The importance and generality of these processes becomes more and more obvious (Rasmussen et al., 2000; Bauer and Mathesius, 2004; von Rad et al., 2008; Teplitski et al., 2011; Hartmann et al., 2014). Hence, considerable effort has been made to examine the influence of AHL on various plants. Since leguminous species are able to mutually associated with bacteria like rhizobia, it is important to compare results gained with them to other, non-legume plants, which can grow in contact with rhizobia, but do not form a symbiotic relationship.

According to our current knowledge, AHL effects on growth parameters, pigment levels or the antioxidant and detoxification enzymes in legumes compared to other plants have not been studied so far. Since AHL or bacteria producing AHL might be used as plant strengthening agents, the background of their potential effect on plants has to be described. Here, our aim was to find out whether and how AHL are able to influence the plants’ pigment status and their antioxidative and detoxifying capacities, because this might lead to a change in resistance toward stress factors like, e.g., UV radiation or applied pesticides. Besides, application of AHL should not impair the plants’ growth and root formation in order not to decrease their performance as crop plants. In consequence, we have examined these parameters as well.

Here, the monocotyledonous crop barley (*Hordeum vulgare* L.) has been chosen for a comparison with the less well-known crop plant yam bean [*Pachyrhizus erosus* (L.) Urban]. This plant with its still under-used potential (Belford et al., 2001) exhibits many interesting features for agricultural cultivation like high-yielding tubers rich in valuable protein and starch, low demand of fertilizer and notable robustness due to a well-equipped detoxification system and the production of a natural insecticide, rotenone (Sørensen, 1996; Belford et al., 2001). *Pachyrhizus* species are naturally associated with bacteria of the genera *Rhizobium* and *Bradyrhizobium* (Sørensen, 1996), producing, amongst others, C6-HSL and C8-HSL (Blosser-Middleton and Gray, 2001). We have recently demonstrated differences in the interaction of barley and yam bean with AHLs in an axenic system. It has been reported that legumes like yam bean are apt to modify AHL, while monocotyledonous plants seem to be less- or incapable of this (Delalande et al., 2005). It is thus very likely that legumes have to ability to modify bacterial QS and subsequent gene expression. Also AHL distribution patterns in plants may vary strongly. While an uptake of C6- and C8-HSL-compounds into the shoots of the monocot plant barley was found, hardly any AHL is taken up, at least in an active form, in *Pachyrhizus* (Götz et al., 2007).

For these experiments, an axenic plant incubation system was chosen to ensure lowest interference with the specific response toward AHL-compounds. The system allowed the investigation of morphological characteristics in young plants. It was of great interest to check possible influence of AHL on parameters like fresh weight, leaf area, pigment concentration, root architecture, or pH in the growth media. In similar axenic growth systems, AHLs could clearly induce changes in growth pattern and gene expression of *Arabidopsis thaliana* (Ortíz-Castro et al., 2008; von Rad et al., 2008). Since bacterial colonization is likely to cause a stress response in the plant, we furthermore focused our research on enzymes involved in oxygen stress response and the detoxification system of plants, which both represent crucial pathways for triggering resistance and finally the plants’ survival. Here, we present new evidence that AHL-compounds influence the enzymatic activities of antioxidative and detoxifying enzymes in roots and shoots of barley and Yam bean plants.

## Materials and Methods

### Axenic Single Plant Growth System

Barley seeds (*H. vulgare* L.), cv. “Barke” (Josef Breun GdbR, Herzogenaurach, Germany) and yam bean seeds [*P.* *erosus* (L.) Urban], cv. “EC 550” (Ebenezer Belford, College of Science, Kwame Nkrumah University of Science and Technology, Kumasi, Ghana) were surface sterilized, grown on NB agar (Merck, Darmstadt, Germany) dishes and seedlings were transferred into an axenic incubation system filled with glass beads and 10 mL pure mineral media (Murashige and Skoog, 1962) as previously described (Götz et al., 2007). To avoid hypoxia the upper 2 cm of the glass bed layer were not filled with liquid media. Axenic barley seedlings were transferred into the sterile system 4 days after germination, yam bean seedlings 7 days after germination. All plants were grown solitary. AHLs (Sigma-Aldrich, Steinheim, Germany) were dissolved in ethanol and added to the mineral media at a final concentration of 10 μM (0.1% ethanol). Control plants were supplied with an equal volume of ethanol.

### Shoot Length and Fresh Weight

Barley shoot length was defined as the upper green plant part beginning at the caryopsis up to the tip of the flag leaf. Yam bean shoot length was measured from the hypocotyl base up to the youngest leaf pair. Leaves and roots of both barley (*n* > 50) and yam bean (*n* = 28) were separated directly after harvest and fresh weight was determined. When *P* ≤ 0.05, differences were considered as statistically significant.

#### Determination of Plant Pigment Content

Extraction of chlorophyll and carotenoids was carried out according to the method described by Lichtenthaler and Buschmann (2001). Crushed, deep-frozen plant material was homogenized in cold 80% (v/v) acetone (Fluka, Taufkirchen) and repeatedly centrifuged and filtered to provide pure extracts. Absorption by pigments was measured at room temperature at the wavelengths 663.2 nm (chlorophyll a), 646.8 nm (chlorophyll b), and 470 nm (carotenoids) using a Beckman DU640-spektrophotometer. The resulting values were calculated to μg pigment per mL plant extract according to the equations provided by Lichtenthaler and Buschmann (2001) and then to μg/g fresh weight.

### Root Parameters

Roots from both plant species were scanned with an Epson 3170 scanner in gray scale mode (600 DPI). The images were then processed using the WinRHIZO software (Régent Instruments, Canada). From each root bundle, the average diameter and the total length of all roots per bundle were calculated and used for calculation of total root surface and total root volume. Furthermore, WinRHIZO allowed the detection of the number of tips per root system, but it was not possible to process data from root hairs.

### Root Exudates and pH

Media were separated from glass beads by suction. At T = 0, and during the experiment, reference media without plant samples were taken and pH immediately determined. Media from plants grown in the axenic system were carefully separated from the plants and processed as described above. pH values of T = 0, controls and plant media samples were compared and statistically evaluated by analysis of variance (ANOVA).

### Protein Extraction

Proteins were extracted using a modified protocol after Menone and Pflugmacher (2005) and Lyubenova et al. (2009). Plants were ground in liquid nitrogen and 1 g of the resulting material was resuspended in 5 mL extraction buffer containing 0.1 M sodium phosphate (pH 6.5), 20% glycerol, 1 mM EDTA, 1% PVP K90 (Sigma-Aldrich, Taufkirchen), 14 mM DTE (Carl Roth GmbH, Karlsruhe), and additionally 0.66% protease inhibitor cocktail set VI (Merck, Darmstadt) to prevent protein degradation. The microsomal protein fraction was homogenized with 20 mM sodium-phosphate buffer pH 7 containing 20% glycerol and 1.4 mM dithioerythritol (DTE). Cytosolic enzymes in the supernatant were stepwise precipitated with ammonium sulfate and re-suspended in 20 mM sodium-phosphate buffer pH 7 and desalted using PD10 columns (GE Healthcare, Freiburg). Protein extracts were stored at -85^∘^C until measurements.

### Enzyme Assays

All assays were performed in microtiter plates using a 96-well-Spectrophotometer (Spectra-Max Plus 384, Molecular Devices, USA) and SoftMax Pro 4.6 for evaluation. All enzyme assays were recorded for 5 min at 25^∘^C. Each reaction contained 5% (v/v) of cytosolic or microsomal protein, if not indicated differently.

#### Glutathione *S*-Transferases (GSTs)

Glutathione *S*-transferase activity was measured using different model-substrates due to the variety of iso-enzymes with different substrate specificity (Lyubenova et al., 2009). All substrates were measured with 1 mM GSH. Fluorodifen conjugation was assayed at 30^∘^C for 1 h according to the method of Scalla and Roulet (2002). Other specifications are depicted in **Table 1**.

**Table 1:** Technical information for the assay of GST activity with different model substrates.

Substrate	Buffer	Wavelength (nm)	Extinction coefficient (mM^-1^ cm^-1^)	Substrate concentration (mM)
Chlorodinitrobenzene (CDNB)	Tris/HCl (0.1 M; pH 6.4)	340	9.6	1
Dichloronitrobenzene (DCNB)	Tris/HCl (0.1 M; pH 7.5)	345	8.5	1
*p*-Nitrobenzylchloride (NBC)	Tris/HCl (0.1 M; pH 6.4)	310	1.8	0.5
*p*-Nitrobenzoylchloride (NBoC)	Tris/HCl (0.1 M; pH 6.4)	310	1.9	0.5
Fluorodifen	Glycin-NaOH (0.1 M; pH 9.5)	400	17.2	0.05

#### Antioxidative Enzymes

Superoxide-dismutase activity was measured as an inhibition of adenochrome generation similar to Kröniger (1994) with an epinephrine test at 480 nm and an extinction coefficient of ε = 4.02 mM^-1^ cm^-1^ (Lara-Nuñez et al., 2006). Catalase was assayed after Verma and Dubey (2003) at 240 nm with an extinction coefficient of ε = 0.036 mM^-1^ cm^-1^. GPOX activity was measured using a test similar to Dixon et al. (1998) by determining NADPH consumption at 340 nm with 10% protein extract with an extinction coefficient of ε = 6.22 mM^-1^ cm^-1^. APOX was assayed according to Vanacker et al. (1998) at 290 nm and ε = 2.8 mM^-1^ cm^-1^. MDHAR was assayed also according to Vanacker et al. (1998) with 10% protein extract at 340 nm with ε = 6.22 mM^-1^ cm^-1^. DHAR was measured according to Vanacker et al. (1998) at 265 nm with ε = 7 mM^-1^ cm^-1^.

All enzyme activities were expressed in katal per weight protein, but later depicted as percent of control and statistically evaluated by ANOVA.

### Determination of Protein Concentration

Protein concentration was determined according to Bradford (1976) by using 10 times diluted Coomassie Brilliant Blue G250 (Fluka, Steinheim) solution and BSA (Sigma-Aldrich, Weilheim) as reference for a standard curve.

### Analysis of AHL Metabolites

Measurements via UPLC and FT-ICR/MS were done as described in Götz et al. (2007). Samples from barley and yam bean root and shoot extracts including samples of control plants were prepared according to the method previously described (Götz et al., 2007). Samples from the mineral medium including T = 0 were purified using solid phase extraction with Bakerbond “light load” C18-columns (J.T. Baker, Netherlands). Aliquots of root and shoot tissue of both plant species were additionally hydrolyzed and the extracts were measured in the “negative-ion” mode. The internally calibrated spectra (up to m/z 6000) were analyzed using the FORMULAE-Software (Research Unit Biogeochemistry and Analytics, Helmholtz Centre Munich). The m/z measured were accepted as AHL derivatives, when the number of N atoms in the molecule was 1, when 3 to 5 O atoms were present and when the number of C atoms was even. The resultant data sheets were aligned with an AHL and HS reference list using software of the Research Unit Biogeochemistry and Analytics (Helmholtz Centre Munich). To avoid false positive results all masses were matched to the lists of the corresponding controls.

## Results and Discussion

### Physiological and Root Parameters

Seventeen days of each 10 μM C6-, C8-, and C10-HSL treatment lead to an increase in barley shoot length of roughly 1 cm, though not significantly (**Table 2**). This observation leads to the question whether this increase in shoot length correlates with an increase in biomass of the fresh shoot or whether it is more likely a result of a change in leaf shape. When barley shoots were checked for fresh weight, it turned out that no statistically significant effect of AHL treatment could be described (**Table 2**), although a slight increase corresponding to elevated shoot length could be determined for plants treated with C8-HSL and C10-HSL.

**Table 2:** Leaf parameters 17 days after AHL application in barley and yam bean.

Leaf parameters	Control	C6-HSL	C8-HSL	C10-HSL
**Barley**
Shoot FW (g)	0.47 ± 0.06	0.46 ± 0.06	0.49 ± 0.06	0.48 ± 0.08
Shoot length (cm)	20.0 ± 3.4	20.8 ± 2.8	21.0 ± 2.1	20.9 ± 3.0
Leaf area (cm^2^)	5.4 ± 1.7	5.5 ± 1.5	5.5 ± 1.5	5.7 ± 1.2
**Yam bean**
Shoot FW (g)	0.36 ± 0.08	0.32 ± 0.09	0.3 ± 0.07^∗^	0.39 ± 0.12
Shoot length (cm)	*Not measured due to herbaceous growth*
Leaf area (cm^2^)	5.5 ± 3.5	4.9 ± 3	5.1 ± 3.1	6.1 ± 4

*^∗^Students *t*-test *P* ≤ 0.05.*

Interestingly, the effects of AHL on yam bean shoot fresh weight were more obvious. Both, C6-HSL and C8-HSL caused a decrease in fresh shoot biomass which was significant for C8-HSL, while C10-HSL treatment tended to increase shoot fresh weight ( **Table 2**).

Root fresh weight more or less followed the pattern observed in the respective shoots (**Table 3**). Substantial differences in root biomass after AHL treatment could not be proven for either barley or yam bean, though decreases in root fresh weight were evident after treatment with C6-HSL, while it tended to increase when the plants were grown supplemented with C8-HSL and C10-HSL. This is in accordance with results obtained from examination of root parameters. Structure and geometry of root biomass was evaluated using WinRHIZO software. Barley root parameters are displayed in **Table 3**. The average total length of root system per plant ranges from 31 to 36 cm, following more or less the tendencies observed in fresh weight measurements. After treatment with 10 μM C8-HSL, total root length was highest, in accordance to the observation that this treatment also led to the highest root fresh weight in barley. Root diameters, surface areas or volumes were not statistically different. This was also the case for the average number of root tips per barley plant, although a stimulation of root tip generation could be observed in AHL-treated barley plants. We also measured the number of root tips per plant. Assuming that each tip represents a single root, the average length per single root was diminished by 0.25–0.3 cm without sufficient significance at *P* < 0.05 (data not shown). Consequently we conclude that AHL treatment indeed may cause stimulation in lateral root formation in barley, as total root length was not affected significantly. In our experimental setup it was not possible to gather information on root hair density. Visual examination though showed an increased root hair density after treatment with C10-HSL in barley. These findings are conclusive with results of Ortíz-Castro et al. (2008).

**Table 3:** Root parameters 17 days after AHL application in barley and yam bean.

Root parameters	Control	C6-HSL	C8-HSL	C10-HSL
**Barley**
Root FW (g)	0.17 ± 0.03	0.16 ± 0.02	0.17 ± 0.03	0.17 ± 0.03
Total root length (cm)	33.8 ± 8.7	31.6 ± 5.7	35.8 ± 7.5	31.2 ± 7.0
Average diameter (mm)	0.80 ± 0.15	0.82 ± 0.15	0.81 ± 0.20	0.85 ± 0.19
Total surface area (cm^2^)	8.09 ± 1.48	7.91 ± 1.18	8.74 ± 4.53	8.44 ± 2.15
Total volume (cm^3^)	0.16 ± 0.04	0.16 ± 0.04	0.18 ± 0.06	0.18 ± 0.07
Number of root tips	14.1 ± 3.8	15.0 ± 3.1	17.0 ± 7.0	14.5 ± 3.8
Average root length/tip (cm)	2.39 ± 0.61	2.11 ± 0.38	2.10 ± 0.44	2.15 ± 0.48
**Yam bean**
Root FW (g)	0.2 ± 0.05	0.18 ± 0.04	0.18 ± 0.04	0.22 ± 0.08
Total root length (cm)	21.9 ± 10	19.9 ± 10.5	21.1 ± 10.1	23.3 ± 13.9
Average diameter (mm)	1.05 ± 0.11	1.00 ± 0.12	1.04 ± 0.16	1.08 ± 0.12
Total surface area (cm^2^)	6.97 ± 2.67	5.93 ± 2.68	6.57 ± 2.69	7.64 ± 4.32
Total volume (cm^3^)	0.18 ± 0.06	0.14 ± 0.06	0.17 ± 0.06	0.20 ± 0.11
Number of root tips	10.0 ± 3.4	9.4 ± 3.4	9.3 ± 3.0	10.3 ± 5.8
Average root length/tip (cm)	2.18 ± 0.99	2.11 ± 1.11	2.27 ± 1.09	2.26 ± 1.35

Yam bean root parameters are shown in **Table 3**. C6-, C8-, and C10-HSL were applied in a concentration of 10 μM like in barley. To reach a comparable developmental stage, yam bean was grown 21 days, thus 4 days longer than barley, in the sterile system. Roots of this plant species had an average total root length of 20–23 cm. Average root diameter was ∼1 mm in both, treatments and controls, without significant differences. The trend of root parameters to decrease after C6-HSL treatment resulted from decreased total root length. Each Yam bean plant had about 10 root tips when harvested. Opposed to barley, no AHL effect on lateral root formation was observed.

**FIGURE 1:**
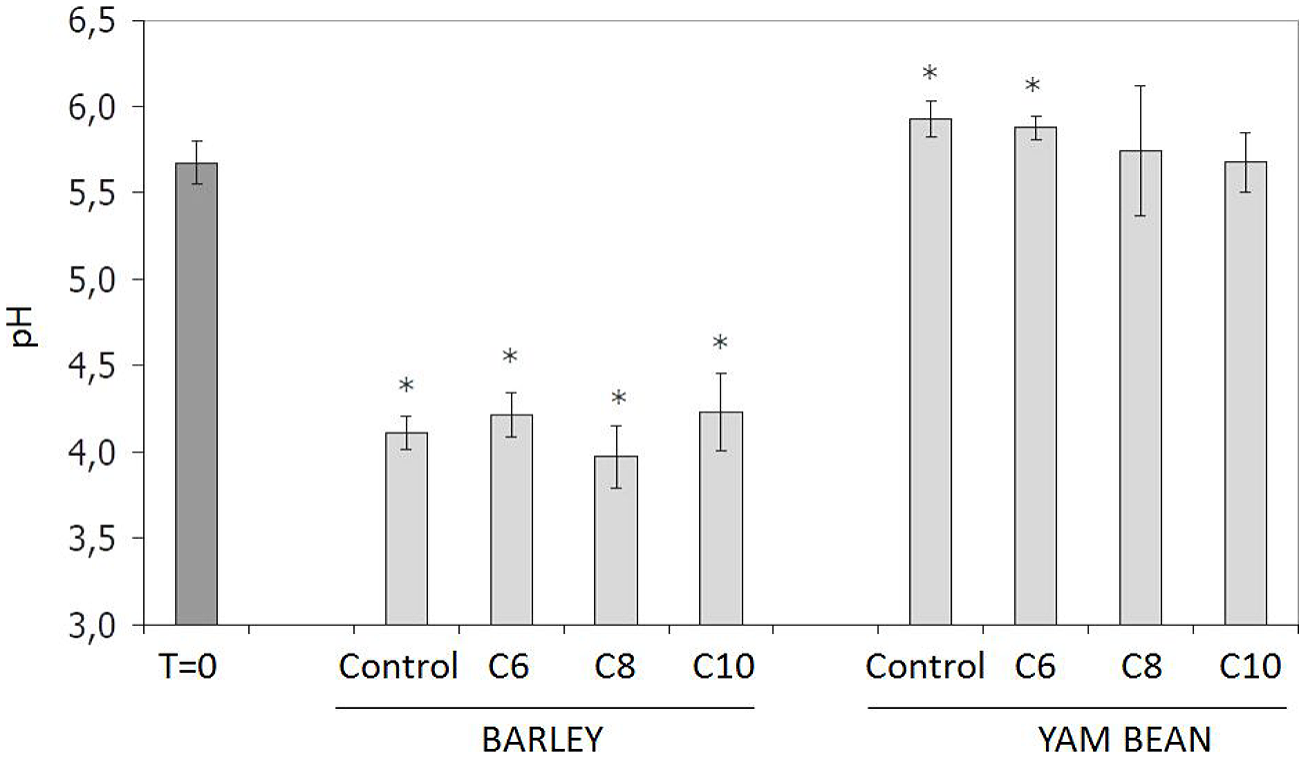
**Influence of plant root exudates and 10 μM AHL application on mineral media pH.** B, Barley; Y, yam bean. Controls contained the same amount of solvent (ethanol) as used in AHL treatments. Asterisks indicate significant differences between T = 0 and time of harvest (ANOVA; ^∗^*P* ≤ 0.05).

Nachin and Barras (2000) reported that chicoree plants infected with the soft rot pathogen *Pectobacterium (Erwinia) chrysanthemi* displayed an elevation of pH surrounding the point of infection. This bacterium possesses a QS-dependent gene regulation system, which involves C6- and C10-HSL in strain Echr 3937 (Barnard and Salmond, 2007). In addition it is known that pH is also highly relevant for AHL stability (Englmann et al., 2007). Thus, the influence of plant growth and AHL application on media pH was examined (**Figure 1**). Samples taken right at the start of the experiment (T = 0) without plant contact displayed an average pH value of 5.7 ± 0.1. Barley caused a significant decrease of media pH to 4.1 ± 0.2. This effect is most probably due to acidic root exudates (Fan et al., 1997). In contrast, no difference in pH value was determined between barley control and the AHL-treated plants. Though AHL treatment did not lead to a significant pH change in barley, it must be noted that the pH reduction in media to pH 4.1 is highly suitable to enhance stability of AHL, which are prone to hydrolyze in alkaline pH ranges (McDougald et al., 2006). This corresponds to our previous finding (Götz et al., 2007) that AHLs in the media are clearly more stable in barley than in yam bean, as well as the observation that yam bean, as opposed to barley, did not cause a pH shift in the culture media. The average pH of the complete yam bean group (control and all treatments) was even found to have significantly increased up to pH 5.8 ± 0.3 at the end of the experiments. C8- and C10-HSL-treated plants were not proven different to T = 0, but in control plants and after C6-treatment an increase in pH was described as significantly different to T = 0. In accordance with the barley experiment, AHL did not influence the pH value when compared to yam bean control plants. Therefore, we conclude that an AHL treatment did not influence the plants’ secretion of pH-active exudates in our setup but might enhance AHL stability in the barley setup by preventing hydrolysis.

### Chlorophyll and Carotenoid Concentration

In barley, bacterial AHL signal substances did not have any influence on chlorophyll and carotenoid concentrations, and also pigment ratios were constant throughout treatments. In yam bean extracts (data not shown) a tendency for a decrease in chlorophyll contents as a consequence of AHL treatment was observed. C10-HSL had a significant effect on yam bean carotenoid levels which were diminished by more than a third compared controls. Consequently, chlorophyll/carotenoid ratio was elevated 1.5-fold compared to control. The biochemical mode of action leading to this decline in carotenoid concentration after C10-HSL application is still under examination. It has been assumed that C10-HSL metabolites, which have recently been traced in axenic plants (Götz et al., 2007), might interfere with carotenoids due to similarities in their long chain aliphatic structure or, on the other hand, might lead to an activation of an enzymatic pathway that subjects carotenoids in yam bean to the same probable degradative process as the long-chain C10- HSL.

### Response of Detoxification Enzymes in Barley

**FIGURE 2:**
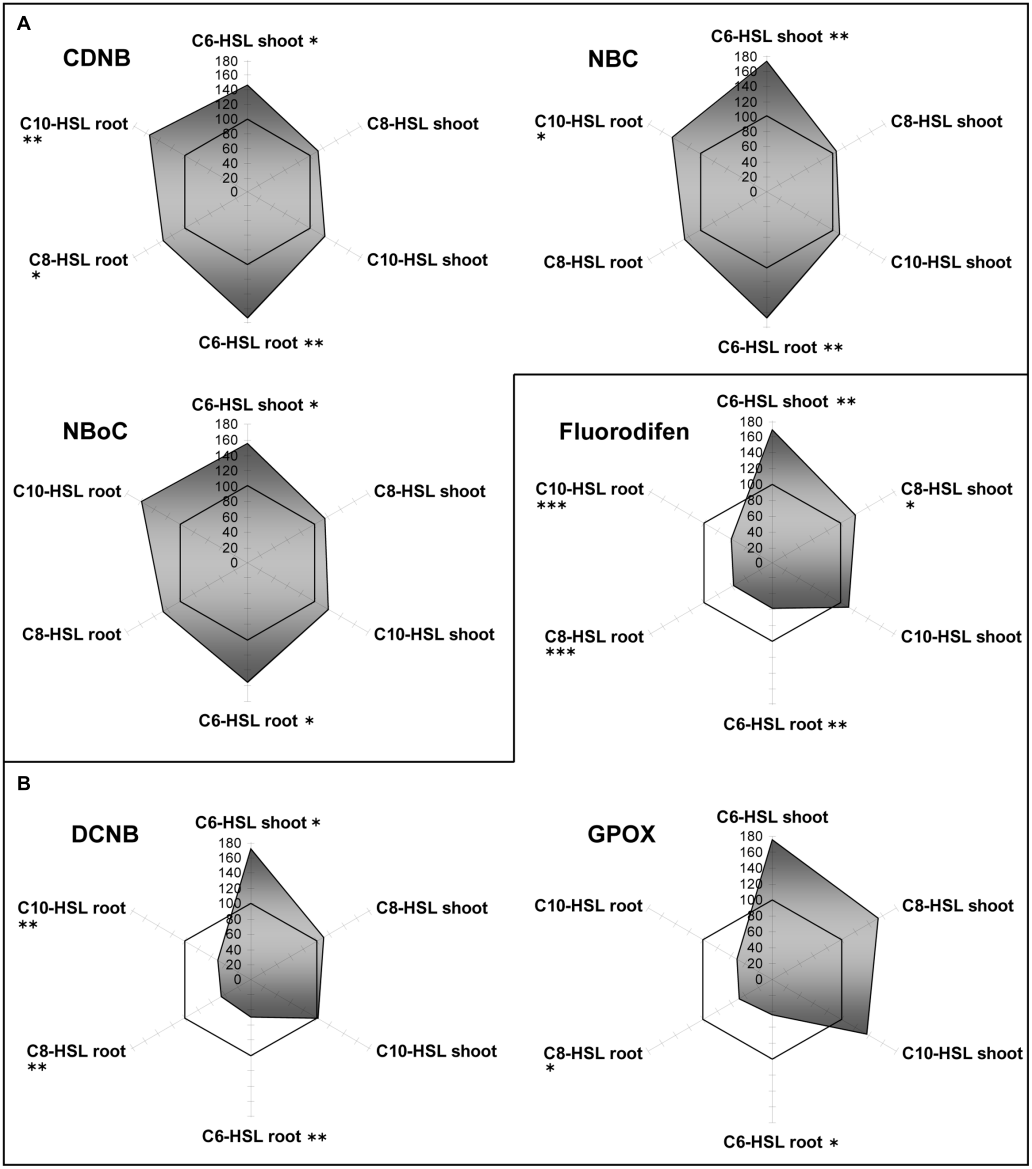
**Radar plot of cytosolic GST enzyme activities in purified protein from barley root and leaf in relation to untreated controls (100% mark).** All measurements were performed at least in triplicate. **(A)** Generally induced GST activities; **(B)** GST inhibition in roots. Asterisks indicate significant differences between the AHL-treatment compared to control treatment (two-sample *t*-test; ^∗^*P* ≤ 0.05; ^∗∗^*P* < 0.01; ^∗∗∗^*P* < 0.001).

Barley cytosolic GST activities were influenced differently by AHL (**Figure 2**). Although the three substrates CDNB, NBOC, and NBC were conjugated at different net rates, the effects of AHL on the GST activity pattern as compared to controls were very similar. In general, effects on roots were stronger than on shoots in barley. C6-HSL elicited significant increase in GST mediated conjugation of the three selected substrates.

At the same time, the conjugation of other GST substrates, namely DCNB and fluorodifen, was strongly inhibited in the roots of all barley plants by the influence of each of the AHLs. In shoots, conjugation remained at control levels under the influence of C8- and C10-HSL, but C6 -HSL caused the same 1.75-fold increase. These results indicate that AHL themselves or a signal connected to AHL sensing in plants, is processed to yield differential induction of GST isoforms. This development of specific GST activity patterns in accordance to the substrate hints toward GST isoform clusters in the plant (Lyubenova et al., 2007). A complementary hypothesis to explain the observed differences between barley roots and shoots might be an effect of direct (root) versus indirect (shoot) exposition toward AHL.

The microsomal membrane fraction of barley roots and leaves contains also several specific GSTs. In microsomes, activities for the conjugation for CDNB, NBC, NBOC, and fluorodifen were detected. Whereas CDNB conjugation remained around control levels throughout, NBC conjugation was increased twice in all tissues. NBOC conjugation seemed to be inhibited in all tissues, except in roots treated with C10-HSL, and fluorodifen conjugation was slightly enhanced in all shoots (Supplementary Figure S1). This proves the prevalence of microsomal GSTs due to a differentiated reaction toward an AHL trigger as compared to the cytosolic GST battery.

**FIGURE 3:**
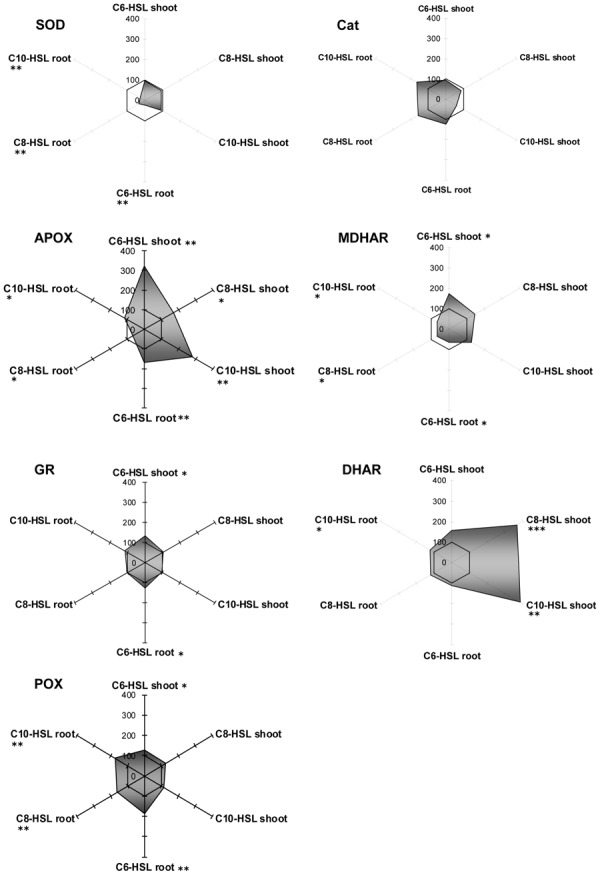
**Radar plot of cytosolic ROS-scavenging enzyme activities in purified protein from barley root and leaf in relation to untreated controls (100% mark).** All measurements were performed at least in triplicate. Asterisks indicate significant differences between the AHL-treatment compared to control treatment (two-sample *t*-test; ^∗^*P* ≤ 0.05; ^∗∗^*P* < 0.01; ^∗∗∗^*P* < 0.001).

Regarding antioxidative enzymes, it is notable that GPOX activity which is subsumed to GSTs by some authors shows exactly the same pattern as observed for GSTs. The three enzymes of the ascorbate dependent defense reactions, namely APOX, DHAR and MDHAR, were inhibited in root tissues, but only to a small extent, whereas they were strongly induced in shoots in an AHL-related reaction. Especially DHAR showed increases up to a factor of 3.5 under the influence of C8- and C10-HSL (**Figure 3**

). Currently it is unknown how this effect in shoots corresponds to AHL treatment.

Generally, SOD and catalase enzymes in plants are denominated as first line of defense proteins against reactive oxygen species generated after pathogen attack (De Gara et al., 2003). Both enzymes reacted on the presence of AHL in barley tissues, but in an unexpected way. Whereas SOD always remained at control levels in shoots, its activity was strongly diminished to about 20% of controls in the treated roots. Not enough, catalase activity was expressed the opposite way. Its activity was lowered in tissues with good SOD activity, but enhanced up to almost twofold in root tissues deprived of SOD. Catalase is an enzyme known for extremely rapid substrate turnover with comparably low substrate affinity. Interestingly, SOD is the other way round with rather low conjugation rates at high substrate affinity. Both enzymes are responsible for the control of reactive oxygen derivates in the tissue, but at different threshold levels, since ROS are not merely destructive agents but also fulfill an important role as a cellular signal substance. Obviously, SOD can react on ROS levels in kind of a “fine tuning” manner, while catalase is more prone to detoxify excess levels of ROS. Since determination of barley and yam bean root ROS levels was not an objective of this work, we can only assume that the shift of SOD toward catalase might be a consequence of a change in oxygen homeostasis in barley roots. However, the question on whether and how AHL might influence plant oxygen turnover is a promising topic for future experiments, which might help to unravel the linkage of enzymatic reactions toward AHL treatment.

### Reaction of Detoxification Enzymes in the Yam Bean

**FIGURE 4:**
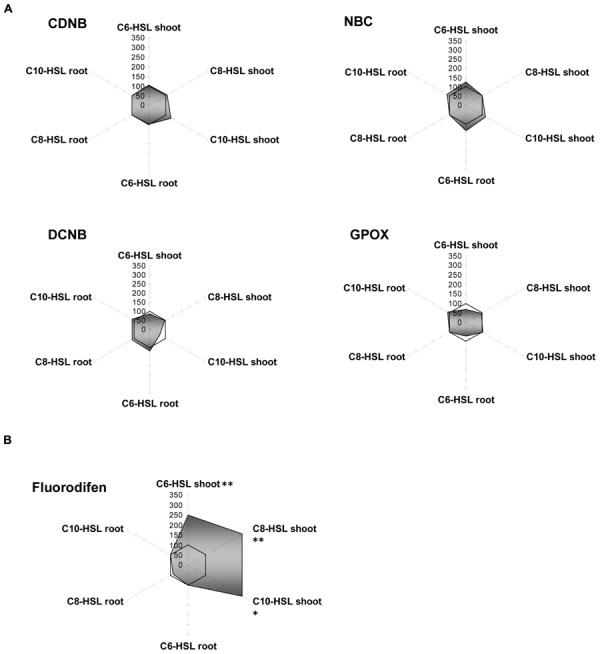
**Radar plot of cytosolic GST enzyme activities in purified protein from yam bean root and leaf in relation to untreated controls (100% mark).** All measurements were performed at least in triplicate. **(A)** No significant effect on GST activity; **(B)** Significant GST induction in shoots. Asterisks indicate significant differences between the AHL-treatment compared to control treatment (two-sample *t*-test; ^∗^*P* ≤ 0.05; ^∗∗^*P* < 0.01; ^∗∗∗^*P* < 0.001).

Yam bean cytosolic GSTs were only poorly influenced (±20%) by an application of the bacterial signal substances (**Figure 4**). As an exception, the GST activity for fluorodifen in the shoot was strongly increased up to threefold under the influence of all AHLs. No NBOC turnover was observed in any tissue, and GPOX activity remained the same in C8-HSL and C10-HSL treated plants, but was inhibited to 60% after C6-AHL application (**Figure 4A**).

Like barley microsomes, the same fraction from yam bean did not respond equally to AHL as the enzymes from the cytosol since different GST clusters are present in microsomes, as discussed before. GST:NBOC activity could be assayed, while it was lacking in cytosolic fractions. For CDNB, NBC and NBOC, reduced enzymatic rates in the shoots were accompanied by an increase in activity in roots. Besides that, fluorodifen turnover did not exhibit any variation after AHL treatment and no DCNB activity was measured (Supplementary Figure S2). Again, as shown in barley, this finding indicates a strategy of concerted reactions in plants due to compartmentation of specific enzyme subsets.

**FIGURE 5:**
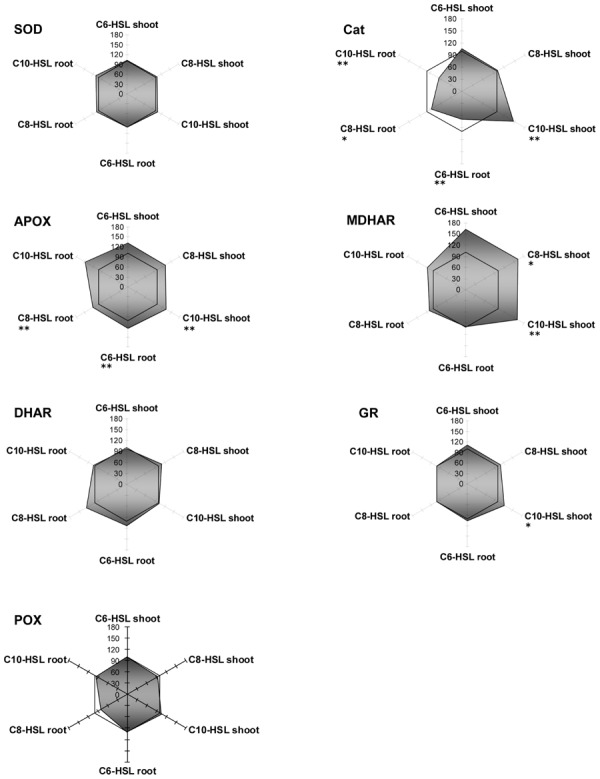
**Radar plot of cytosolic ROS-scavenging enzyme activities in purified protein from yam bean root and leaf in relation to untreated controls (100% mark).** All measurements were performed at least in triplicate. Asterisks indicate significant differences between the AHL-treatment compared to control treatment (two-sample *t*-test; ^∗^*P* ≤ 0.05; ^∗∗^*P* < 0.01; ^∗∗∗^*P* < 0.001).

Amongst the antioxidative enzymes, SOD, DHAR, and GR activities were not altered in any tissue of yam bean. Catalase showed slight inhibition in the roots, whereas APOX activity increased by 25% in all tissues, and MDHAR activity increased by 50% in the shoots only, both under all three AHL treatments (**Figure 5**).

Overall, yam bean detoxification enzymes seemed to be hardly impressed by the AHL signals. This could be due to different strategies of uptake and degradation of AHLs in both plants. Bacteria from the genera *Rhizobium*, *Sinorhizobium,* or *Bradyrhizobium* are known to produce a range of different AHLs playing a role in several of the probably most complex signal cascades yet known (Marketon and González, 2002; Wisniewski-Dyé and Downie, 2002; Pongsilp et al., 2005). Since legumes live in very close relationship with these bacteria (Wisniewski-Dyé and Downie, 2002) and would therefore naturally and continuously be exposed to AHL signal substances originating from their nitrogen-fixing bacterial partner, it can be assumed that they have learnt to “deal” with these compounds during co-evolution with the producers.

### Analysis of AHL Breakdown Products

Previously we could show via UPLC measurements that the plant dependent degradation of C6-, C8-, and C10-HSL in the mineral media in barley amounts 17, 27, and 35%, respectively, whereas in yam bean it is more than doubled (49, 67, and 71%, respectively). We could also confirm via FTICR/MS that all three AHL molecules were present in root tissue of barley and yam bean, whereas only C6- and C8-HSL could be detected in barley shoots and C6-HSL was the only detected AHL entering into yam bean shoots (Götz et al., 2007).

In the above mentioned previous publication we have thus addressed the question how AHLs are taken up and translocated and how they might be degraded. However, these experiments do not provide information about the quality of the signal detected or the grade of breakdown of the original compound, respectively. Furthermore, it is highly interesting to find out in which plant tissue or at which stage AHLs are metabolized. Therefore, experiments were carried out using FTICR/MS to screen for early AHL metabolites in root and shoot tissue of both plant species. Besides hydrolysis of the lactone ring the decrease in length of the acyl side chain or the introduction of reactive groups in unsaturated AHLs can be considered as potential start points of AHL degradation.

Separation of the acyl side chain from the lactone ring through, e.g., aminoacylases could not be detected, probably because of the relatively low mass of the lactone ring (m/z 102) that was below the effective range of FTICR/MS detection (m/z 150-10.000) and due to the fact that fatty acids in plant extracts are not suited as targets for metabolite analysis, because of the difficulty to assign them correctly to AHLs. Besides, lactonase can be characterized as a key player in AHL degradation according to our findings and those from Delalande et al. (2005). Due to the higher AHL depletion in the media of yam bean plants and the difference of AHL detection in the shoot between both plants the probability of a distinct enzymatic degradation strategy of AHLs in both plants seems logical. Delalande et al. (2005) also reported AHL degradation in *Lotus corniculatus* by either lactonase or acylase. Also, the influence of barley root exudates on pH and subsequent AHL hydrolyzation as described above should be kept in mind.

**FIGURE 6:**
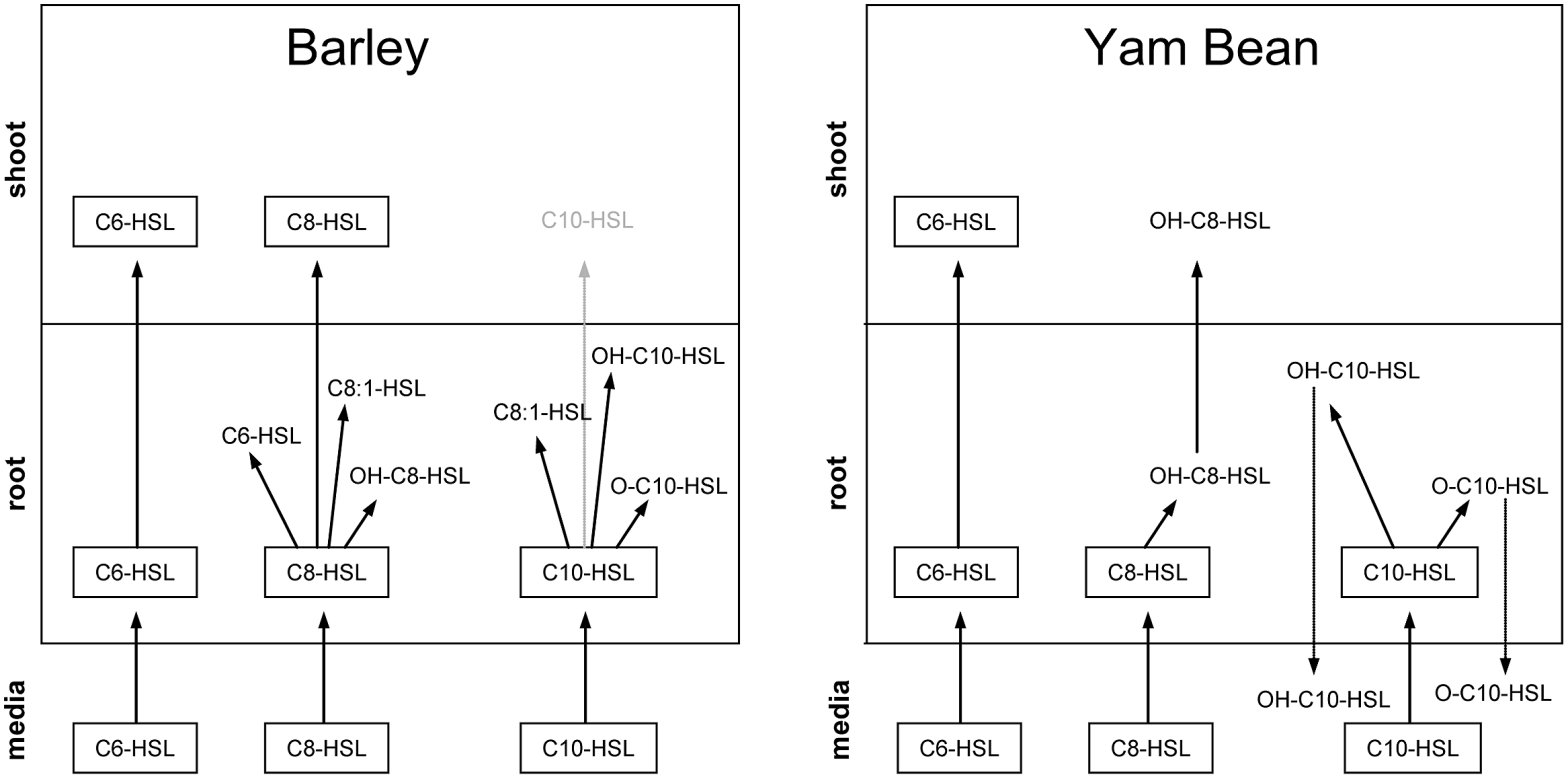
**Schematic image of AHL compounds, their distribution and their corresponding metabolites in the mineral media and in root and shoot tissue of barley and yam bean detected via UPLC and FTICR/MS.** The C10-HSL in barley shoots could only be detected after radioactive labeling and is therefore shown in light gray.

Because of the large number of potential metabolites we decided to restrict our search to very early metabolites with a structure that could undoubtedly be assigned to the applied AHL (see **Figure 6** for an overview). We were unable to find any candidate metabolite in barley roots upon C6-HSL application but in both C8- and C10-HSL treated barley plants we found a metabolite that was shortened by one C2 group at the acyl side chain and the hydroxyl form of both AHLs (**Table 4**). In the C8-HSL treated plants we also detected a C8-metabolite with an unsaturated side chain and in C10-HSL treated plants we found an oxo-C10 metabolite.

**Table 4:** *N*-acyl-homoserine lactone metabolites in barley and yam bean root and shoot extracts.

Root	Barley	rel. Int.	^13^C	Yam Bean	rel. Int.	^13^C
						
C6-HSL	n.d	–	–	n.d	–	–
						
C8-HSL	C6	1.41E+07	det.			
	C8:1	8.51E+06	det.			
	OH-C8	1.77E+07	det.	OH-C8	5.60E+06	n.d.
						
C10-HSL	C8:1	5.40E+06	n.d.			
	O-C10	6.55E+06	n.d.	O-C10	3.27E+06	n.d.
	OH-C10	1.75E+07	det.	OH-C10	5.69E+07	det.

**Shoot**	**Barley**	**rel. Int.**	**^**13**^C**	**Yam bean**	**rel. Int.**	**^**13**^C**

						
C6-HSL	n.d.	–	–	n.d.	–	–
						
C8-HSL	n.d.	–	–	OH-C8	5.04E+06	det.
						
C10-HSL	n.d.	–	–	n.d.	–	–

*n.d., not detected; det., detected; rel. int., relative intensity; ^13^ C= identical structure with a ^13^ C isotope.*

In roots of yam bean plants we were again unable to detect any metabolite after C6-HSL application nor was a shortened AHL metabolite present. Only the hydroxy-form of both C8- and C10-HSL and the keto-form of C10-HSL were discovered. While we could not detect any metabolite in barley shoots, one metabolite, precisely the hydroxy-form of C8-HSL was found in yam bean shoots.

No metabolites were detectable in the mineral medium except for yam bean medium where the same metabolites were found as in roots, namely, hydroxy-C10, and oxo-C10-HSL (data not shown).

Taken together, it is obvious that most of the primary metabolites are detectable in plant roots, which indicates that AHL breakdown probably starts very early after uptake of the compound directly in root tissue. Further metabolites may then be formed in both root and shoot of the plant or small breakdown products might be translocated, but root tissue seems to be the crucial initial stage of AHL metabolization. A very important finding in this context is the almost complete lack of AHL metabolites in mineral media. Only in C10-HSL treated yam bean, a metabolite was found in media, but it is not possible to decide whether this product was formed in media or whether it was transported downstream in root tissue. According to these data we can state that AHL breakdown is most probably going on inside the plants’ root and not outside the plants via, e.g., secreted factors.

Another important finding is the connection of AHL acyl chain length and predisposition for metabolization. Our experiments clearly prove that the longer the side chain, the more rapidly or easily the AHL gets degraded. C6-HSL, for example, is hardly degraded but readily transported into all plant parts, while C10-HSL is almost broken down completely before it has even the chance to enter the shoot in its initial form. In this context the effect of the plant species selected for the experiments also is explicitly important: in yam bean, which is naturally exposed to AHL, the metabolization of the bacterial signal proceeds faster and probably more precisely than in barley which does not undergo bacterial symbiosis.

Finally, there’s an open question regarding the mode of AHL degradation. We have strong hints for an involvement of enzymes like lactonase and/or acylase. Possible candidates forming hydroxylated metabolites of AHL might also be Phase 1 detoxification enzymes like cytochrome P450-monooxygenases, which are able to transform hydrophobic substrates into more reactive and lipophilic products. The topic remains an interesting field for further screens like for later AHL breakdown products or the enzymatic mode of degradation.

## Conclusion

Although we could observe a certain tendency of growth change after AHL application in both plant species those were not significant despite a large number of replicates. Similarly the content of chlorophylls and carotenoids did not change except in yam bean after C10-HSL application. Hence, the influence of bacterial AHL signaling compounds on growth and photosynthesis of the investigated plant species must be considered as very low. This contrasts the finding in *A. thaliana* (von Rad et al., 2008) where significant stimulation of root growth was observed upon addition of C6- and C8-HSL at similar concentrations. In tomato plants (MicrotomR), the same AHL-compounds stimulated the expression of pathogen defense genes and made the inoculated plants more resistant toward the leaf pathogen *Alternaria alternata* (Schuhegger et al., 2006). Whilst this was not found in *A. thaliana* or barley, when treated with C6-, C8-, and C10-HSL compounds (von Rad, personal communication), Schikora et al. (2011) clearly demonstrated that the length of the acyl moiety and the functional group at the γ-position specify the plant’s response to AHLs. Specifically, oxo-C14-HSL-treated *Arabidopsis* plants were more resistant toward bacterial pathogens.

Thus, plants seem to respond differently to AHL-compounds, which points to different receptors or signaling cascades. However, until now, no specific AHL-receptor has been identified in plants. Nevertheless we observed a distinct change in the pattern of enzyme activity after AHL treatment. Surprisingly, the activity of some enzymes increased up to 3.5-fold yet 17d after AHL treatment. The reaction of GSTs and ROS scavenging enzymes upon AHLs is mostly tissue specific. An equal level or a reduction of enzyme activity in root tissues and an increase in shoot tissues could be observed in a large group of enzymes. In a second group of enzymes there was either no change in enzyme activity or the change was not tissue specific, but more or less equal. In a few cases a compound specific change in enzyme activity was observed, e.g., GPOX activity in yam bean after C6-HSL application (**Figure 3**), however, the majority of the enzymes measured react solely on the AHL application and did not “sense” the different length of the acyl side chain.

In accordance with these findings of apparent influences of AHL and possibly also their metabolites on enzymatic activities in different tissues, we were able to detect AHLs and several metabolites in roots of both plants, while in barley shoots only C6- and C8-HSL and in yam bean only C6-HSL and OH-C8-HSL as a metabolite of C8-HSL could be detected via UPLC and FTICR/MS (see **Figure 6** and also Götz et al., 2007). However, in previous experiments with tritium labeled C8-and C10-HSL and barley plants, we observed an uptake of both compounds into the shoot (Sieper et al., 2013). Both HSLs were also systemically transported into the shoot 2 h after application. In addition, a newly established detection method with mAbs allowed the first detection of a systemic transport of long-chain HSLs in plants. The coupled use of different HSL detection methods demonstrated that the uptake and transport of HSLs into barley does not occur passively, but relies, at least partially, on active processes in the plant.

Since only low concentrations of C10-HSL related metabolites were found in shoots of barley, while in yam bean only C6-HSL was found and since the plant dependent decrease of AHLs in the mineral medium in yam bean is more than twice as high as in barley it is reasonable to assume that yam bean is able to metabolize and degrade AHLs at an earlier stage and to greater extent than barley. This might be an explanation why detoxification and plant stress related enzymes react quite dramatically in barley upon AHL treatment while in yam bean as a whole they seem to be rather unimpressed. However, the presence and action of an endogenous plant signaling compound which plays a role in the reaction of the shoots cannot be excluded and is subject to ongoing studies.

## Conflict of Interest Statement

The authors declare that the research was conducted in the absence of any commercial or financial relationships that could be construed as a potential conflict of interest.

## Supplementary Material

The Supplementary Material for this article can be found online at: http://www.frontiersin.org/journal/10.3389/fpls.2015.00205/abstract

## Abbreviations

AH: N–acyl–D/L–homoserine lactone
APOX: ascorbate peroxidase
C6–HSL: N-hexanoyl–D/L–homoserine lactone
C8–HSL: N–octanoyl–D/Lhomoserine lactone
C10–HSL: N–decanoyl–D/L–homoserine lactone
CAT: catalase
DHAR: dehydroascorbate reductase
GPOX: glutathione peroxidase
GR: glutathione reductase
GSH: glutathione
GST: glutathione S–transferase;
MDHAR: monodehydroascorbate reductase
SOD: superoxide dismutase
