# Supplementary material for: Influence of bacterial N-acyl-homoserine lactones on growth parameters, pigments, antioxidative capacities and the xenobiotic phase II detoxification enzymes in barley and yam bean
Source: Front Plant Sci. 2015 Apr 10;6:205. doi: 10.3389/fpls.2015.00205 (PMC4392610; doi:10.3389/fpls.2015.00205)
Supplement: Supplementary file 1 [file Image_1.PDF]

Supplemental Figure 1

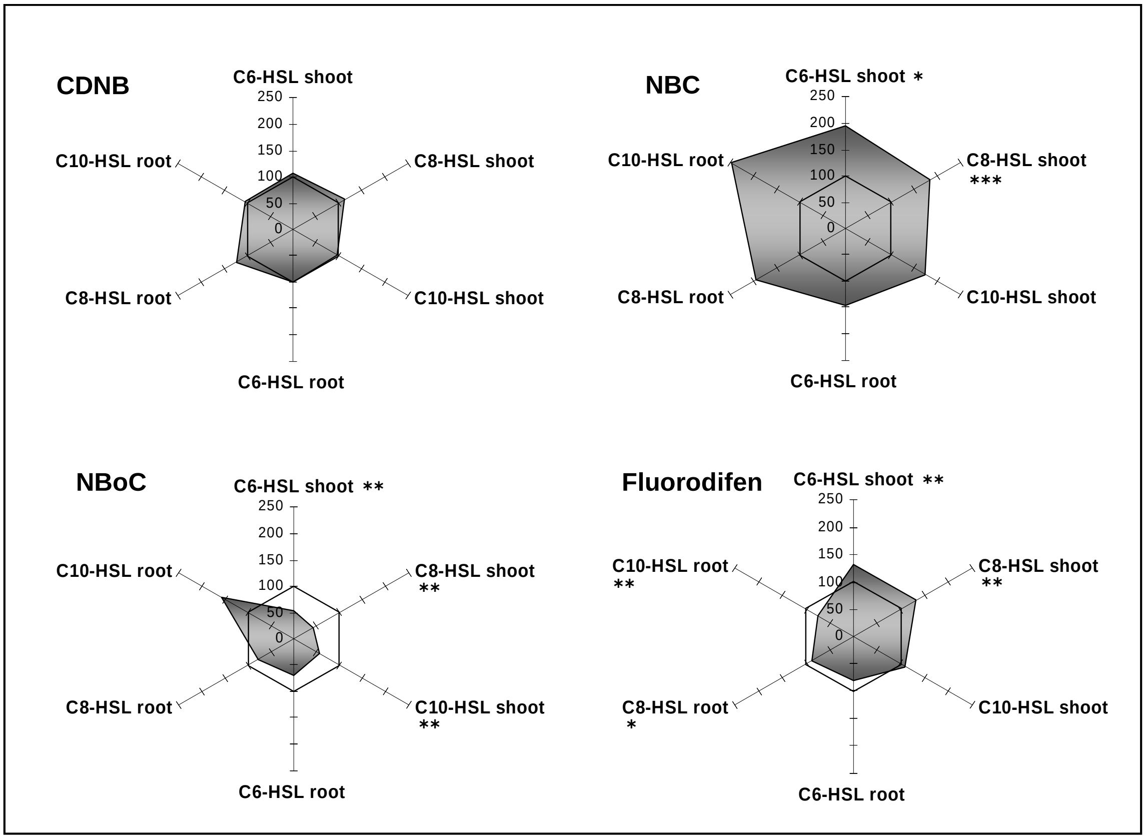

Supplemental Figure 1:

Radar plot of microsomal GST activities in barley root and leaf extracts in relation to untreated controls (100% mark). All measurements were performed at least in triplicate.
