# Supplementary material for: Influence of bacterial N-acyl-homoserine lactones on growth parameters, pigments, antioxidative capacities and the xenobiotic phase II detoxification enzymes in barley and yam bean
Source: Front Plant Sci. 2015 Apr 10;6:205. doi: 10.3389/fpls.2015.00205 (PMC4392610; doi:10.3389/fpls.2015.00205)
Supplement: Supplementary file 2 [file Image_2.PDF]

Supplemental Figure 2

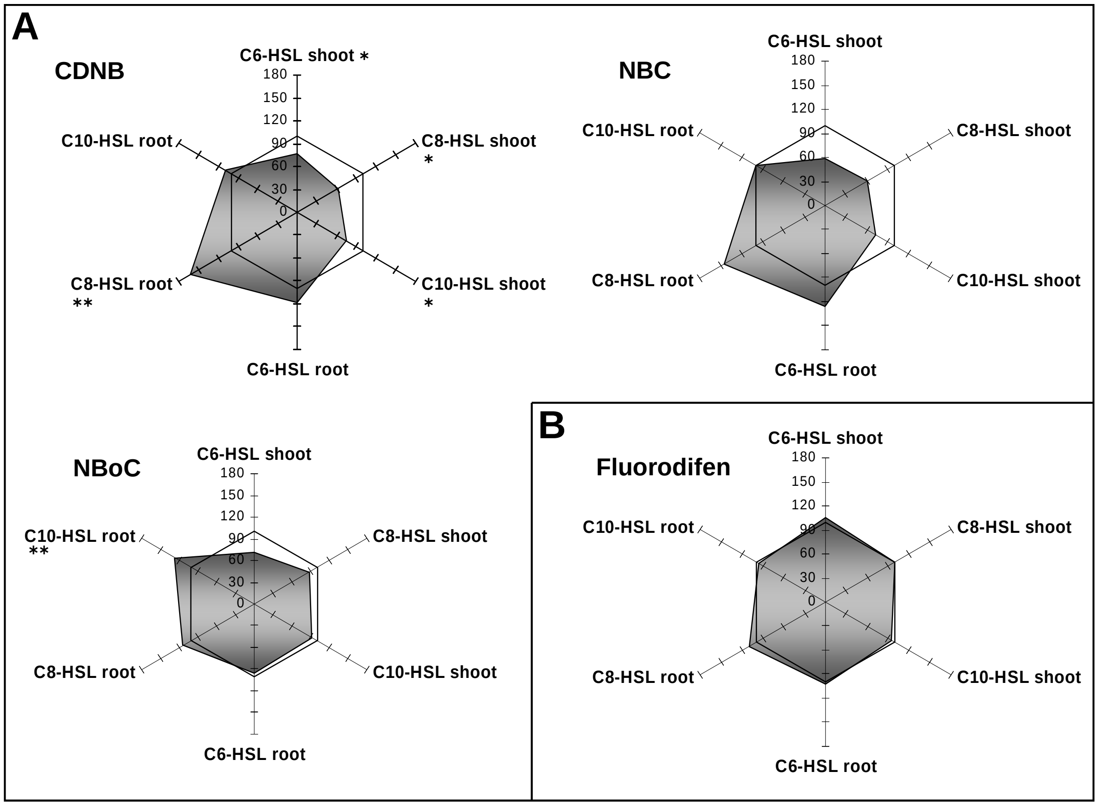

Supplemental Figure 2:

Radar plot of microsomal GST activities in yam bean root and leaf extracts in relation to untreated controls (100% mark). All measurements were performed at least in triplicate.
